# The “Phagocytic Synapse” and Clearance of Apoptotic Cells

**DOI:** 10.3389/fimmu.2017.01708

**Published:** 2017-12-04

**Authors:** Nicole D. Barth, John A. Marwick, Marc Vendrell, Adriano G. Rossi, Ian Dransfield

**Affiliations:** ^1^MRC Centre for Inflammation Research, Queen’s Medical Research Institute, University of Edinburgh, Edinburgh, United Kingdom

**Keywords:** macrophage, phagocytosis, apoptotic cells, cell–cell interactions, phagocytic receptor, phosphatidylserine, opsonin

## Abstract

Apoptosis and subsequent phagocytic clearance of apoptotic cells is important for embryonic development, maintenance of tissues that require regular cellular renewal and innate immunity. The timely removal of apoptotic cells prevents progression to secondary necrosis and release of cellular contents, preventing cellular stress and inflammation. In addition, altered phagocyte behavior following apoptotic cell contact and phagocytosis engages an anti-inflammatory phenotype, which impacts upon development and progression of inflammatory and immune responses. Defective apoptotic cell clearance underlies the development of various inflammatory and autoimmune diseases. There is considerable functional redundancy in the receptors that mediate apoptotic cell clearance, highlighting the importance of this process in diverse physiological processes. A single phagocyte may utilize multiple receptor pathways for the efficient capture of apoptotic cells by phagocytes (tethering) and the subsequent initiation of signaling events necessary for internalization. In this review, we will consider the surface alterations and molecular opsonization events associated with apoptosis that may represent a tunable signal that confers distinct intracellular signaling events and hence specific phagocyte responses in a context-dependent manner. Efficient molecular communication between phagocytes and apoptotic targets may require cooperative receptor utilization and the establishment of efferocytic synapse, which acts to stabilize adhesive interactions and facilitate the organization of signaling platforms that are necessary for controlling phagocyte responses.

## Introduction

Elimination of injured or metabolically stressed cells in multicellular organisms is controlled *via* engagement of apoptotic programs together with efficient tissue clearance mechanisms (1–3). Phagocyte/apoptotic cell interactions also initiate anti-inflammatory reprogramming that regulates inflammation and immunity (4). Deficient clearance of apoptotic cells contributes to the development and/or exacerbation of many autoimmune and inflammatory diseases [reviewed in Ref. (5)].

**Figure 1:**
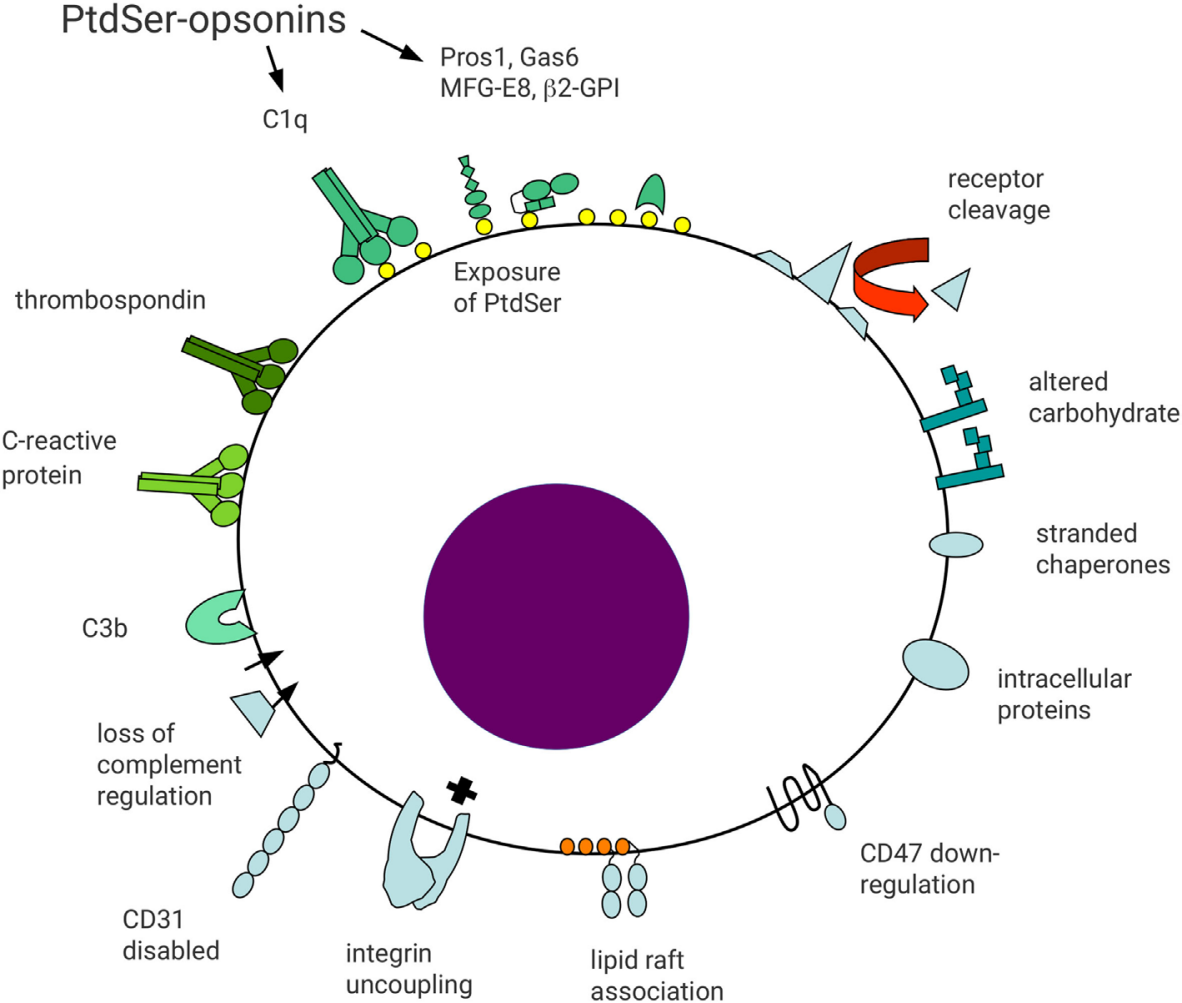
Schematic representation of surface molecular changes associated with apoptosis. Reduced cell surface molecule expression may occur through metalloprotease-mediated proteolytic shedding. Reduced receptor expression may act to limit apoptotic cell function and generate a distinct cell surface profile from viable cells. For example, reduced expression of CD47 or disabled CD31 may lead to loss of signals preventing phagocytosis. In addition, loss of integrin regulation may result in functional uncoupling in apoptotic cells, leading to cell detachment. Altered association of cell surface molecules with lipid rafts may alter functional activity, including gain-of-function of some receptors, e.g., FcγRIIa on myeloid cells. Altered carbohydrate processing may result in reduced sialic acid exposure and appearance of accessible mannose residues. Downregulation of complement regulatory molecules (e.g., CD55 and CD46) may lead to opsonization with complement components including C3b. Exposure of anionic phospholipids, including phosphatidylserine (PtdSer), allows binding of a broad range of opsonins to apoptotic cells. Protein S and Gas6 bind to PtdSer in a Ca^2+^-dependent manner, whereas milk fat globule EGF-factor 8 (MFG-E8) binds independent of Ca^2+^. Other less well defined apoptotic cell surface changes may allow binding of other opsonins including thrombospondin, C-reactive protein, and surfactant protein A. Finally, proteins with intracellular localizations may appear on the surface of apoptotic cells, including heat-shock proteins and calreticulin. Apoptotic cell surface molecules are shaded blue whereas apoptotic cell opsonins are shaded green.

The diversity of molecular pathways mediating recognition and phagocytosis of apoptotic cells (efferocytosis) reflects the fundamental importance of this process (4). There are several mechanisms by which viable cells avoid phagocytosis (6). However, altered plasma membrane lipid composition (7, 8) and/or oxidation status (9), together with changes in cell surface molecule repertoire and patterns of glycosylation (10) termed “apoptotic cell associated molecular patterns” (11) (Figure 1), allow phagocytes to distinguish viable and apoptotic cells. Here, we consider the formation of an “efferocytic synapse” and assembly of molecular platforms that facilitate phagocytosis and subsequent signaling events.

## Phosphatidylserine (PtdSer) as a Ligand for Apoptotic Cell Recognition

A near universal membrane alteration associated with apoptosis is the caspase-dependent exposure of PtdSer on the outer leaflet of the plasma membrane (12–14) *via* the XK-related protein 8 (15). Exposure of PtdSer affects the biophysical characteristics and organization of the plasma membrane through recruitment of proteins to PtdSer-enriched regions *via* electrostatic interactions (16). Phagocytes express transmembrane receptors that bind PtdSer directly, e.g., brain-specific angiogenesis inhibitor-1 (BAI-1) (17) and stabilin-2 (18). In addition, soluble molecules such as transthyretin-like protein TTR-52 (19), milk fat globule EGF-factor 8 (MFG-E8) (20, 21), protein S (Pros1), Gas6 (22) and C1q (23) also bind to (and opsonize) PtdSer, providing a scaffold for phagocyte recognition *via* a diverse array of counter-receptors.

Phagocytes fail to engulf viable cells that expose low levels of PtdSer during activation (24–26) or when PtdSer exposure is induced by overexpression of a phospholipid scramblase, transmembrane protein 16F (TMEM16F) (27), suggesting that additional signals are necessary to initiate efferocytosis. A critical threshold of PtdSer exposure on the cell surface may be required to trigger efferocytosis (28). For example, recognition of PtdSer *via* T-cell immunoglobulin and mucin-domain-containing molecule (TIM)-4 was dependent on ligand density, allowing phagocytes to distinguish between high and low level PtdSer exposure (28). Further modifications of PtdSer during apoptosis, e.g., oxidation or formation of lyso-PtdSer (29) may also be important.

## Cell Surface Receptor Alterations Associated with Apoptosis

Apoptosis-dependent loss of cell surface receptors or appearance of “new” molecules may confer recognition by phagocytes. For example, signaling *via* Signal regulatory protein-α (SIRPα) inhibits myosin-II-mediated phagocytosis (30). Downregulation of ligands for SIRPα, e.g., CD47 (31), from the surface of apoptotic cells would be predicted to promote efferocytosis (32, 33). SIRPα-mediated signaling has also been reported to be triggered by binding of surfactant protein (SP)-A and SP-D to phagocytes. However, SP-A may have a dual role in regulation of phagocytosis as binding to apoptotic cells/debris results in promotion of phagocytosis *via* a calreticulin/CD91-mediated pathway (34). Early experiments identified a unique charge-sensitive mechanism for apoptotic cell recognition (35). The cell glycocalyx provides a negative surface charge “repulsive” force that counters cell–cell interactions (36). Loss of N-terminal sialic acid and exposure of mannose and fucose moieties during apoptosis reduces electrostatic forces that counter phagocyte recognition (37–39). In addition, the surface charge of apoptotic cells is further altered by reduced expression of heavily sialylated proteins (e.g., CD43, CD45, and CD162) (37). Consistent with this suggestion, removal of sialic acid from the cell surface by neuraminidase treatment enhances phagocytosis (37, 39).

Apoptosis is associated with loss of expression of complement regulatory proteins such as CD46/CD55 (40, 41). As a result, deposition of complement may occur, providing a cue for recognition by phagocytes. Additional signals for phagocytosis may occur as a result of exposure of intracellular proteins such as calreticulin (42) and annexin I (43). Following binding to PtdSer on apoptotic cells, oxidation of Pros1 induces oligomerization, which promotes Mer-dependent phagocytosis (44). Similarly, altered glycosylation of membrane proteins or oxidization of low-density lipoprotein-like moieties on apoptotic cells (8) may also contribute to the specific recognition by phagocytes. In addition to increased expression of ligands for phagocytic receptors on apoptotic cells, patching and/or clustering of surface molecules may also have important consequences for triggering phagocyte responses. Clustering might occur through specific association with membrane microdomains. For example, FcγRIIa redistributes to membrane microdomains during neutrophil apoptosis (45). In addition, specific proteolysis of adhesion molecules (e.g., CD62L) (46, 47) and uncoupled β_2_ integrin-mediated adhesion (47) during apoptosis is likely to provide additional molecular cues for phagocytosis.

## Phagocyte Molecules that Mediate Apoptotic Cell Recognition

Phagocytes are capable of direct recognition of PtdSer exposed on the apoptotic cell surface. BAI-1 binds PtdSer *via* thrombospondin (TSP) type 1 repeats present in the extracellular domain (48). Binding induces the formation of a trimeric complex of BAI-1 with the Rac-GEF ELMO and DOCK180 that promotes subsequent engulfment of apoptotic cells (17, 49). This pathway is homologous to the genetically defined pathway for removal of apoptotic cells in *Caenorhabditis elegans* (Ced2-CrkII, Ced5-DOCK180, Ced10-Rac, and Ced12-ELMO) (50).

Phosphatidylserine is also recognized by the CD300 family of molecules with an extracellular IgV-like domain and intracellular adaptor molecule binding sites (51). CD300b localizes to phagocytic cups and binds DAP12, activating Syk and PI3K/Akt to promote phagocytosis (52). Stabilin-2 binds PtdSer and also lacks direct signaling activity (18). However, the cytoplasmic domain of stabilin-2 can interact with GULP to facilitate phagocytosis (53). GULP also binds to NPxY motifs present in the cytoplasmic domains of CD91/LRP (low-density lipoprotein receptor-related protein) and the *C. elegans* scavenger receptor Ced-1 (54). In contrast, TIM-4 confers Ca^2+^-dependent PtdSer-dependent apoptotic cell recognition, but lacks intracellular signaling potential (55). Thus, TIM-4 functions cooperatively with other receptors that trigger apoptotic cell internalization.

Indirect recognition of apoptotic cells by phagocytes is also achieved by phagocyte receptors that bind to soluble apoptotic cell opsonins. In *C. elegans*, TTR-52 bridges apoptotic cell-exposed PtdSer to phagocyte Ced-1 (19), which together with Ced-6 initiates rapid and efficient engulfment of apoptotic cell corpses by neighboring cells. This module of proteins (Ced1-MEGF-10; Ced6-GULP; and Ced7-ABCA1) has been defined genetically in *C. elegans* (50). Pros1 and Gas6 contain a Gla-domain that binds PtdSer in a Ca^2+^-dependent manner (22), bridging to the Tyro3/Axl/Mer receptor tyrosine kinases that signal particle internalization *via* intrinsic kinase activity (56). By contrast, MFG-E8 binds PtdSer in a Ca^2+^-independent manner and bridges to phagocyte integrins α_v_β_3/5_ *via* arginine–glycine–aspartic acid (RGD) peptide motifs in the C1 and C2 domains (21). TSP-1 also bridges apoptotic cells *via* the phagocyte integrin α_v_β_3_ and CD36 (57, 58).

## Cooperative Receptor Utilization in Phagocytosis of Apoptotic Cells

Phagocytes in different tissue settings or microenvironments express distinct repertoires of efferocytic receptors. Whether a single phagocytic cell utilizes multiple receptor pathways to recognize and internalize a single apoptotic target is not clear. However, the distinct molecular requirements for the capture and subsequent internalization may require that multiple receptors are involved (42). In addition, the complex topology of apoptotic cell surface molecules and co-opsonization of PtdSer with different proteins may determine the spectrum of signal transduction pathways engaged, controlling internalization and subsequent phagocyte responses in a context-dependent manner.

Tethering of IgG-opsonized particles to FcγR occurs at 4°C (59) whereas internalization requires cytoskeletal reorganization and metabolic activity (60). Similarly, apoptotic cells can also be tethered by phagocytes *via* Mer at low temperature (61). However, the avidity of low affinity receptors is influenced by receptor density and rapid lateral movement of receptors to facilitate target capture (62, 63). Receptor mobility is controlled by cytoskeletal constraint, association with membrane lipid microdomains and/or other membrane proteins (64). For example, cytoskeletal-associated CD44 restricts membrane lipid and receptor motility *via* interactions with hyaluronan, forming a glycosaminoglycan barrier that reduces binding of phagocytic targets (36). Interestingly, CD44 cross-linking with antibody augments macrophage phagocytosis of apoptotic cells, possibly as a result of changes in cytoskeletal regulation (65, 66). Phagocytic targets are bound at dynamic extensions of phagocytic cells, including filopodia and membrane ruffles (67), and receptor aggregation is required for orchestration of cytoskeletal alterations necessary for internalization [see Ref. (68) for a comprehensive review of cytoskeletal regulation in phagocytic synapses].

In contrast with IgG or complement attached to components of the microbial cell wall, molecules on the surface of apoptotic cells may exhibit unrestricted lateral mobility as a consequence of proteolytic cleavage of actin during apoptosis (69). Thus, there may be key mechanistic differences between efferocytosis and FcγR-dependent phagocytosis. Engagement of freely mobile molecules by phagocyte receptors may lead to assembly of receptor microclusters and significantly impact upon phagocytosis (33). Alternatively, opsonization of apoptotic cells may result in formation of immobile molecular complexes [like annexin V (70, 71)] that promote redistribution of phagocytic receptors necessary for signaling of internalization.

## Molecular Segregation and the Formation of a Phagocytic “Synapse”

The cellular contact between phagocyte and apoptotic cells has parallels with those of antigen-specific T and B cells with antigen presenting cells that leads to establishment of an immunological synapse (72). This specialized intercellular contact zone stabilizes adhesion and facilitates efficient molecular communication following antigen-specific interactions. A number of different biophysical factors (including receptor density, ligand-binding affinity, molecular dimensions, and interactions with cytoskeletal elements) all contribute to the dynamic redistribution of adhesion and signaling receptors into distinct regions in the plasma membrane (73, 74).

Following phagocyte contact with IgG-coated surfaces or on supported lipid bilayers, formation of FcγRII nanoclusters suggests activation-driven organization of receptor redistribution (75). FcγR clusters are localized in front of ruffles on extending pseudopods, with rapid recruitment of Syk to advancing pseudopods and subsequent retrograde movement toward the cell center (76). PI3K co-localized with actin around FcγR clusters, suggestive of signal propagation from FcγR and consistent with PI3K-dependent control of actin cytoskeletal rearrangements (76).

Although phagocyte contact with apoptotic cells has not been examined with the high-resolution imaging techniques used in T cell–APC interactions, molecular segregation may also be a key feature of the formation of efferocytic synapses (see Table 1). For example, exclusion of phosphatases (e.g., CD45) from the immune synapse is a critical early event in the initiation of T cell receptor-mediated phosphorylation of Zap70 and Lck (77). Changes in the distribution of CD45 may also represent a general feature of membrane alterations that control signaling events associated with phagocytosis. The C-type lectin containing receptor for β-glucan, Dectin-1, mediates the recognition and phagocytosis of yeast particles. During Dectin-1-mediated phagocytosis, exclusion of CD45 and the receptor type protein tyrosine phosphatase CD148 from the nascent phagocytic cup (78) is important for signaling associated with particle internalization (79). Redistribution of phosphatases in the phagocyte membrane would likely be necessary for internalization of apoptotic cells.

**Table 1:** Comparison of phagocytic synapse formation in FcγR-mediated and apoptotic cell phagocytosis.

	FcγR phagocytosis	Apoptotic cell phagocytosis
Receptor signaling^a^	ITAM or ITIM adaptors Src/Syk kinases PI3K	BAI-1:G protein-coupled (DOCK/ELMO Rac-GEF) Tyro3/Axl/Mer: receptor tyrosine kinase, PI3K, Rac Stabilins:GULP adaptor? CD300:ITIM or DAP12 adaptors α_v_ integrins (Rac?) TIMs:suppression of Src, none?
Ligand restraint	Restricted mobility	Not known Some ligands restrained by oligomerization?
Phosphatases	CD45/CD148 excluded from contact	Not known
Role of integrins	Formation of exclusion zone	Not known/co-receptors: α_v_β_3_ and CD36 α_v_β_5_ and Mer β_1_ integrins and TIM-4
Receptor nanoclusters	Yes	Not known
Molecular dimensions	7–10 nm (FcγR) 15 nm (IgG) +Antigenic target	Very small (3–4 nm), e.g., CD300 to very large (40–50 nm), e.g., SCARF1/C1q

*^a^See Elliott et al. (80) for a comprehensive overview of receptor signaling*.

*ITIM, immunoreceptor tyrosine-based inhibition motif; ITAM, immunoreceptor tyrosine-based activation motif; BAI-1, brain-specific angiogenesis inhibitor-1*.

Analysis of the molecular basis of redistribution of CD45 following ligation of FcγR suggests non-linear pattern with formation of an exclusion barrier which restricts access of CD45 to the contact site (79). CD45 is a relatively rigid molecule that extends axially from the plasma membrane approximately 20 nm (81). Similar to the molecular redistribution that occurs during immune synapse formation, exclusion from phagocytic synapses was dependent on the axial molecular dimensions of CD45. In experiments using chimeric constructs in which the extracellular portion of CD45 was replaced by either CD43 (similar length) or CD2 (shorter length), the CD43/CD45 molecule was excluded from the phagocytic synapse, whereas CD2/CD45 was not (79). A requirement for integrins and cytoskeletal regulation was shown to be necessary to establish a CD45 exclusion zone that extended beyond the IgG layer (79). These results suggest that integrin-mediated contact between phagocyte and phagocytic targets facilitates engagement of phagocytic receptors at low ligand densities or when binding to larger particles.

For apoptotic cell recognition, it is intriguing that integrins have been proposed to act cooperatively with other receptors to mediate phagocytosis. For example, α_v_ integrins and CD36 are both required for the recognition of TSP-1 bound to apoptotic cells (58). Similarly, phagocytosis of apoptotic cells *via* TIM-4 requires β_1_ integrins and activation of integrin-dependent signaling involving Src family kinases and FAK (82). Furthermore, interactions between the β_5_ integrin and stabilin-2 were found to promote phagocytosis of apoptotic cells (83). Similarly, co-expression of α_v_β_5_ with Mer increased activation of Rac-1, cytoskeletal regulation and the phagocytosis of apoptotic cells (84). Integrins can directly mediate the recognition of apoptotic cell opsonins, for example, RGD-dependent binding of MFG-E8 (21) or fibronectin (85). However, phagocytosis of apoptotic targets is increased following macrophage adhesion to extracellular matrix *via* β_1_ integrins (86) and is compromised following exposure to oxidized extracellular matrix molecules (87). We would speculate that, as for FcγR-mediated phagocytosis, integrin signaling can regulate cytoskeletal organization and facilitate tethering/phagocytosis of apoptotic cells with low-level opsonization (36).

For human macrophage-like cells, CD47 expression acts to limit phagocytosis of IgG-opsonized erythrocytes (30). CD47 binds to phagocyte SIRPα, resulting in recruitment to the phagocytic synapse, decreasing the accumulation of non-muscle myosin IIa and levels of tyrosine phosphorylation (30). Localization of SIRPα to the site of cell contact would recruit inhibitory tyrosine phosphatases such as SHP-1 *via* ITIM motifs present in the SIRPα cytoplasmic domain (88). In the absence of CD47 or when CD47 was blocked with antibody, phagocytosis was increased. Specific membrane receptors are organized into protein islands in unactivated T cells that subsequently coalesce as a consequence of T cell receptor-mediated signaling (89). The membrane distribution of SIRPα and FcγR during phagocytosis has been further analyzed using super-resolution microscopy. When macrophages were plated onto poly-l-lysine-coated slides, molecular clusters containing both FcγRI and SIRPα were observed (75). These molecules were found to segregate into discrete nanoclusters when macrophages were plated onto IgG. Interestingly, IgG promoted the formation of concentric rings of FcγRI and FcγRII, with FcγRI redistributing more rapidly (<10 min). Similar results were obtained when macrophages interacted with IgG in a supported lipid bilayer. When recombinant CD47 was included into the supported lipid bilayer, segregation of FcγR and SIRPα and the formation of concentric rings of FcγR were blocked. Thus co-localization of SIRPα and FcγR inhibits cellular activation following FcγR ligation, whereas segregation of these two molecules leads to activation (75). We would speculate that efferocytic receptors would also be present in dynamically regulated nanoclusters in the phagocyte membrane.

## Molecular Dimensions and Apoptotic Cell Recognition

**Figure 2:**
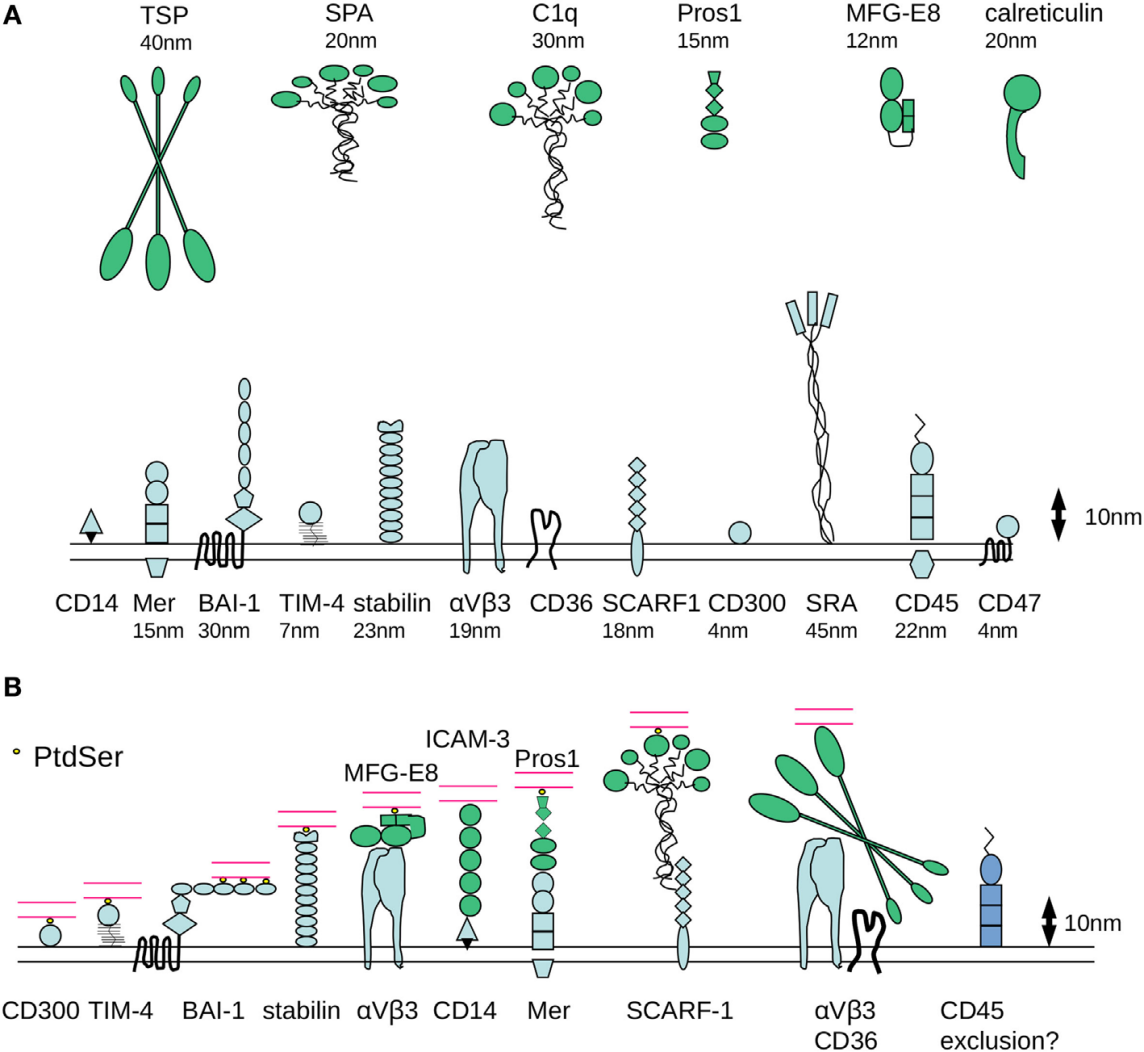
Molecular dimensions of proteins involved in phagocytosis of apoptotic cells. **(A)** Some of the major families of receptors that have been identified as having a role in the phagocytosis of apoptotic cells are depicted, drawn approximately to scale based on available published crystallographic/NMR or cryo-electron microscopy data for various protein domains. One assumption made is that the molecules have a relatively rigid structure, although it is possible that flexibility would considerably alter axial length. There are considerable differences in the dimensions of apoptotic cell opsonins and in the receptors that mediate binding of phosphatidylserine (PtdSer) binding (either directly or indirectly). **(B)** Example receptor–counter-receptor pairings are shown to illustrate the likely differences in intermembrane “working” distance during phagocytosis, particularly for the molecules involved in the recognition of PtdSer (shown in yellow). Phagocyte receptors are shown in blue, opsonins are shown in green, and the apoptotic cell membrane is shown in red.

Size-dependent redistribution of molecules within the phagocyte membrane may represent an important organizing principle for the assembly of molecular platforms that are essential for signaling the cytoskeletal alterations required for the internalization of apoptotic cells. Estimation of the molecular dimensions of receptors involved in efferocytosis using published structural data (51, 90–106) reveals considerable differences in axial dimensions (Figure 2A).

First, it is notable that CD300 (single Ig-like domain) and TIM-4 (single Ig-like domain with a potentially rigid mucin-like stalk), which mediate direct recognition of PtdSer, are predicted to span a relatively short intermembrane distance between phagocyte and target (4 and 7 nm, respectively, Figure 2B). By contrast, stabilin-2, which also binds to PtdSer, is a much longer molecule extending some 23 nm. Assuming a degree of structural flexibility, BAI-1 may be capable of functioning in a broad range of intermembrane distances. The extracellular region of BAI-1 contains 5 TSP type 1 repeats (around 5 nm in size) with LPS and PtdSer binding motifs, which together with the GAIN/HBD regions could extend to ~33 nm from the plasma membrane. Following initial tethering of PtdSer by the N-terminal TSP repeat, BAI-1 could align parallel to the apoptotic cell surface as additional TSP repeats become ligated (Figure 2B). Nevertheless, it seems likely that a degree of molecular segregation would be required for these different receptors to be involved in apoptotic cell uptake on the same phagocyte.

Second, receptors such as SR-A or LRP are predicted to be highly extended molecules (~40–50 nm). In addition, some of the well characterized opsonins for apoptotic cells are extremely large. For example, C1q is approximately 30 nm and TSP-1 is 40–50 nm. Together with the relatively large counter receptors for these opsonins [e.g., SCARF1 for C1q (107) and α_v_β_3_ for TSP], the predicted intermembrane contact distance would likely be incompatible with those of BAI-1, α_v_β_5_/MFG-E8, or Mer/Protein S. One possibility is that these extended structures are efficient in the initial capture of apoptotic targets, facilitating subsequent engagement of receptor/counter-receptor pairs that span a smaller intermembrane distance and are influenced by electrostatic repulsion between cells.

Third, receptors of the Tyro3/Axl/Mer family, BAI-1, and the integrin α_v_β_5_ are able to initiate intracellular signaling that controls particle internalization. If there are parallels with immunological synapse formation, these receptors might be expected to become localized at the center of a contact zone. However, the intermembrane distance for TAMs and integrins to engage their counter-receptors (~30 nm) is considerably larger than that of the immunoreceptors that are responsible for signaling during the establishment of the immunological synapse (~14 nm for the TcR/MHC class II and co-stimulatory receptors such as CD80/CD28). We would speculate that the organizing principles for immune and efferocytic synapses would be different due to the distinct requirements for signal propagation following cognate interaction of receptors. In an immune synapse, delivery of a signal that controls T cell proliferation or target cell killing requires maintenance of intercellular adhesion and redistribution of antigen-specific recognition molecules to the contact zone. By contrast, an efferocytic synapse would likely be a more dynamic structure that facilitates particle tethering and allows dynamic regulation of cytoskeletal organization as particle internalization proceeds. As discussed earlier, the localization of receptors involved in phagocytosis may involve initial tethering mediated by larger molecules as a prerequisite for engagement of smaller PtdSer binding receptors. The mechanisms for exclusion of phosphatases from an efferocytic synapse might also be distinct.

Finally, although phagocytosis of intact apoptotic targets is readily observed *in vitro*, the tight apposition of the plasma membranes of phagocytes and apoptotic targets in certain tissues *in vivo* may require a mechanistically distinct clearance process. For example, in the retina clearance of the outer segments of photoreceptors by retinal pigment epithelial cells has been likened to a phagocytic “pruning” of the photoreceptor outer segments (108). The molecular basis of removal of photoreceptor outer segments by retinal pigment epithelial cells involved Mer, Pros1 and Gas6, and the integrin α_v_β_5_ (108–110). Electron microscopy analysis of retinal cell architecture reveals the exquisitely close contact between RPE cells and photoreceptors (111). Elegant *in vivo* imaging studies have revealed the diurnal exposure of PtdSer in localized a manner, which then triggers the “pinching off” of the distal tips of the photoreceptors by the adjacent RPE cells (112). The exposure of PtdSer on viable photoreceptors may have some parallels with a process termed phagoptosis (113) in which viable cells are recognized by phagocytes. However, during phagoptosis, recognition triggers apoptosis in the target cell (114). Although similar molecular pathways are involved in the recognition of PtdSer exposure on viable cells (e.g., MFG-E8, stabilins, α_v_ integrins, and Mer) (115), the intercellular communication events that are involved are likely to be distinct from those required for efferocytosis.

In summary, the establishment of an efferocytic synapse may be required for efficient recognition of apoptotic cells by phagocytes. Cooperativity of receptor engagement may act to facilitate and stabilize adhesive interactions and lead to the assembly of signaling platforms that ultimately determine phagocyte responses to apoptotic cell binding and internalization.

## Author Contributions

ID wrote the article and generated the figures. NB, JM, MV, and AR wrote the article and critically appraised the figures.

## Conflict of Interest Statement

The authors declare that the research was conducted in the absence of any commercial or financial relationships that could be construed as a potential conflict of interest.
